# Phytochemical Screening, Pharmacognostic Characterization, Antioxidant Activity, and Hepatoprotective Effects of *Abroma augustum* (L.) L.f. on Human Hepatocellular Carcinoma (HepG2) Cells and Goat Liver Homogenate

**DOI:** 10.3390/antiox14040472

**Published:** 2025-04-15

**Authors:** Sandipan Das, Tanushree Deb, Filomena Mottola, Nithar Ranjan Madhu, Yogisharadhya Revanaiah, Israel Maldonado Rosas, Sarbani Dey Ray, Shubhadeep Roychoudhury

**Affiliations:** 1 Department of Life Science and Bioinformatics, Assam University, Silchar 788011, India; 2 Department of Medicine, Silchar Medical College, Silchar 788014, India; 3 Department of Environmental, Biological and Pharmaceutical Sciences and Technologies, University of Campania Luigi Vanvitelli, 81100 Caserta, Italy; 4 Department of Zoology, Acharya Prafulla Chandra College, New Barrackpore 700131, India; 5 ICAR-Krishi Vigyan Kendra Hailakandi, ICAR Research Complex for NEH Region, Hailakandi 788152, India; 6 Citmer Reproductive Medicine, Mexico City 11520, Mexico; 7 Department of Pharmaceutical Sciences, Assam University, Silchar 788011, India

**Keywords:** Devil’s cotton, methanolic extract, phenolics, flavonoids, oxidative stress

## Abstract

*Abroma augustum* (L.) L. f. is characterized by its fibrous structure, spiny trichomes, and distinctive leaf formations, which collectively contribute to its unique morphology and potential medicinal applications. This study aims to investigate the phytochemical constituents and elucidate the pharmacognostic and physicochemical characteristics of the stem bark powder, including evaluation of its antioxidant capacity and hepatoprotective effects against carbon tetrachloride (CCl_4_)-induced hepatotoxicity in both in vitro and ex vivo experimental models. Comprehensive phytochemical screening identified 50 distinct phytochemicals, including a range of alkaloids, flavonoids, terpenes, phenolics, and coumarins, among others. The extract displayed substantial solubility, with total phenolic and flavonoid content quantified as 12.32 ± 0.01 mg/g and 42.14 ± 3.5 mg/g, respectively. The antioxidant activity revealed IC_50_ values obtained from 2,2-diphenyl-1-picrylhydrazyl (DPPH), ferric reducing antioxidant power (FRAP), and 2,2′-azinobis-(3-ethylbenzothiazoline-6-sulfonic) acid (ABTS), measured at 214.007 µg/mL, 132.307 µg/mL, and 45.455 µg/mL, respectively. Additionally, the methanolic extract exhibited significant hepatoprotective properties, with observable reductions in lipid peroxidation and decreased concentrations of liver damage biomarkers (ALT, AST, and LDH) in both HepG2 cells and goat liver homogenate. Future investigations are warranted to elucidate the underlying mechanisms of these effects, including histopathological examinations and biochemical assays, followed by the administration of plant methanolic extracts.

## 1. Introduction

Medicinal plants have been employed as natural remedies for centuries. In recent years, there has been a notable increase in global research focused on these plants, underscoring their considerable potential within traditional healthcare systems [1,2]. The advancement of plant-based medicinal systems has led to the emergence of several established traditional medicinal practices, such as Ayurveda and Unani within the Indian subcontinent; Traditional Chinese Medicine in China; and numerous indigenous healing systems across Africa, North America, and South America. These systems demonstrate a comprehensive understanding of the pharmacological properties of plants and their incorporation into integrated health practices, underscoring the significance of cultural context in the evolution of medicinal knowledge [3,4,5]. A substantial portion of the global population depends on medicinal plants as a primary healthcare resource. This reliance is notably intensified in developing nations, where the demand for these resources escalates. This dependency underscores the vital role that medicinal plants play in conventional healthcare practices [5,6]. Even in developed nations, traditional plant-based remedies have gained popularity and are used as complementary or alternative medicines [7]. Approximately 2000 plant species are utilized in Ayurveda for their therapeutic properties, while the Chinese Pharmacopoeia encompasses approximately 5700 traditional medicines, most of which continue to be employed in contemporary practice. The increasing interest in traditionally used plants is attributed to their notable medicinal properties, which have garnered significant attention in academic and pharmacological research. This resurgence underscores the importance of integrating traditional knowledge with contemporary scientific approaches to enhance therapeutic efficacy and promote sustainable practices in medicine [8]. The World Health Organization (WHO) endorses the integration of traditional plant-based medicines into national healthcare systems, supporting their use and promoting public trust in these interventions [9].

*Abroma augustum* (L.) L.f., also called Devil’s cotton, is a perennial medicinal plant belonging to the Malvaceae family. This species is distributed globally in tropical and subtropical climates, with its primary indigenous range encompassing India, Southeast Asia, Northern Australia, and the Solomon Islands [10]. This plant is used in traditional medicinal systems of Bangladesh and India to treat various illnesses. The pharmacological potential of *A. augustum* (L.) L.f. is closely linked to the cultural beliefs and medicinal practices surrounding its use [11]. Previous studies have effectively demonstrated the curative properties of various plants of the Malvaceae family [12,13], highlighting the need for further investigation into the specific pharmacological significance of *A. augustum* (L.) L.f. Continued exploration in this area could yield valuable insights into its therapeutic applications and efficacy. Standardized drugs have undergone verification of their identity, along with assessments of their quality and purity. Given the commercialization of plant-based medicinal formulations, implementing quality control criteria for various medicinal plants used in traditional medicine is vital. Pharmacognostic investigations are crucial in elucidating the plants’ key morphological, anatomical, and chemical characteristics. Therefore, conducting pharmacognostic investigation is essential for precise identification and ensuring the safety and efficacy of plant-based products in conventional and modern healthcare systems [14]. The acceptance, efficacy, and safety of plant-based medicines contribute to their overall quality and effectiveness. Inadequate quality control measures may compromise the efficacy and safety of these products, potentially leading to adverse health outcomes for consumers. Consequently, drug standardization has become a pivotal area of research, focusing on addressing issues of adulteration, among others. Therefore, the demand for standardized medications that ensure consistent quality is critical for public health [15].

Medicinal plants are a rich source of natural antioxidants, primarily due to their bioactive compounds, including phenolics, flavonoids, tannins, alkaloids, terpenes, and saponins [16]. Secondary metabolites, especially phenolics and flavonoids, exhibit significant antioxidant properties by scavenging reactive oxygen species (ROS) and reactive nitrogen species (RNS). These mechanisms contribute to the mitigation of oxidative stress-related diseases, including cancer, rheumatism, chronic bronchitis, asthma, renal failure, diabetes, aging, and neurodegenerative disorders [17]. Additionally, they can act as reducing agents and metal chelators [18]. Consequently, assessing the antioxidant potential of such plants is imperative to evaluate their pharmacological efficacy against various diseases. Nonetheless, selecting an appropriate methodology to measure antioxidant capacity is essential. The most frequently employed assays used to determine the antioxidant potential of plant extracts include 2,2-di-phenyl-1-picrylhydrazyl (DPPH), 2,2′-azinobis-(3-ethylbenzothiazoline-6-sulfonic) acid (ABTS), and ferric-reducing antioxidant power (FRAP) [19].

The liver is a vital organ within the human body, responsible for a variety of critical physiological functions. These include the biosynthesis of bile, protein production, nutrient storage, regulation of hemostatic factors, maintenance of hormonal balance, and detoxification of both exogenous and endogenous harmful substances [20]. Liver diseases can culminate in severe conditions such as hepatosis, hepatitis, and, ultimately, cirrhosis. The etiology of liver dysfunction encompasses a range of factors, including viral infections, drug-induced toxicity, excessive consumption of alcohol, autoimmune conditions, genetic predisposition, and metabolic disorders [21]. Liver diseases are prevalent and recognized as major public health concerns. Conventional pharmacological treatments for liver diseases often exhibit substantial side effects, including low in vivo stability, reduced hepatic distribution, and limited target efficacy, in addition to disrupting the normal function of other essential organs. Consequently, there is an increasing focus on phytotherapy for liver disorders [22].

Plant polyphenols are gaining significant attention due to their hepatoprotective effects, which include scavenging free radicals [23], alleviating inflammation [24], regulating liver enzymes [25], and preventing apoptosis and fibrosis [26]. For example, polyherbal formulations utilizing bioactive fractionation of three traditionally utilized plants—*Butea monosperma*, *Bauhinia variegata*, and *Ocimum gratissimum*—have exhibited significant efficacy against acute hepatotoxicity induced by paracetamol [27]. Bioactive compounds derived from plants, including andrographolide, curcumin, kutkoside-1, sauchinone, phyllanthin, berberine, embelin, resveratrol, and acteoside, also exhibit significant pharmacological properties [28] and are thus recognized as effective hepatoprotective agents. Glycyrrhizin, liv-52, and silymarin have, therefore, been marketed following clinical investigations [28].

The present study aimed to screen the phytochemicals and standardize the physicochemical and pharmacological properties to ensure the quality and efficacy of bioactive compounds of *Abroma augustum* (L.) L.f., thereby establishing a robust foundation for future research and clinical trials. Additionally, evaluating the antioxidant potential through DPPH, FRAP, and ABTS assays, alongside investigating the hepatoprotective effects of the plant methanolic extract (PME) using human hepatocellular carcinoma cells (HepG2) and goat liver homogenate, will facilitate the integration of this medicinal plant into contemporary healthcare practices. In this study, we utilized the Soxhlet apparatus for PME preparation, recognized as one of the most effective methods for extracting phytocompounds from plant samples, offering scalability and reproducibility [29]. This research also has the potential to identify new antioxidant and hepatoprotective biomolecules. Ultimately, the present study may pave the way for further research to validate the traditional therapeutic claims of *Abroma augustum* (L.). L.f.

## 2. Materials and Methods

### 2.1. Phytochemical Screening Using Liquid Chromatography–High-Resolution Mass Spectrometry (LC-HRMS)

A liquid chromatography–high-resolution mass spectrometry system equipped with a quadrupole time-of-flight mass spectrometer (QTOF-MS; Model G6530C, Agilent Technologies, Santa Clara, CA, USA) coupled to a dual electrospray ionization source was utilized to identify the phytochemicals present in the PME. The instrument was operated in positive ionization mode. Chromatographic separation was achieved using a reversed-phase C18 column (Agilent ZORBAX Eclipse Plus C18, 2.1 × 100 mm, 1.8 µm particle size) maintained at 40 °C under gradient elution. The mobile phases consisted of solvent A: 95% water with 0.1% formic acid, and solvent B: 5% acetonitrile with 10% water and 0.1% formic acid. The total flow rate was maintained at 400 μL/min. Samples were introduced via injection mode with needle wash enabled to minimize carryover. An injection volume of 10.00 μL was used for all analyses. The system was equilibrated for 5 minutes before each run. The mass spectrometer settings were optimized for metabolite detection. Raw data files were processed using MZmine 2.5 software [30]. Continuous data acquired in positive ion mode were processed. Data processing, specifically feature extraction, using MZmine included the following steps: data import, MS peak detection, chromatogram construction, chromatogram deconvolution, isotope grouping, peak alignment, MS row filtering, and gap filling. Accurate mass values of the fragments obtained in the same ionization mode were matched with the database, set with a tolerance of 5 ppm.

### 2.2. Preparation for Pharmacognostic Studies

**Figure 1:**
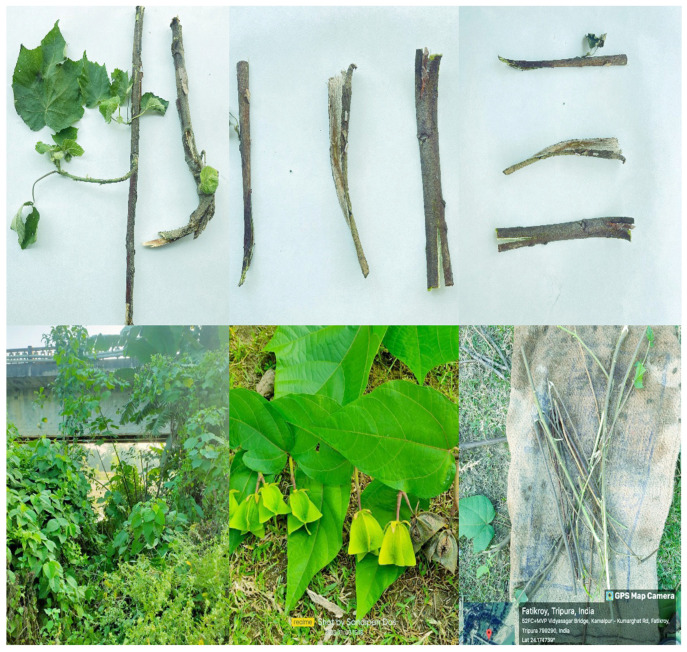
Collection and preparation of *Abroma augustum* (L.) L.f. stem barks from Fatikoroy, Unakoti, Tripura, Northeastern India. Processing protocol entails the systematic collection of disease-free stem barks, followed by drying and subsequent pulverization for additional analytical investigations.

*A. augustum* (L.) L.f. samples were collected from Fatikroy, Unakoti District, Tripura, Northeast India, under conditions free from pathogens and diseases (Figure 1). The specimens were systematically identified and deposited at the Botanical Survey of India, Shillong (Reference number BSI/ERC/Tech/2024–25/372). Following the collection, all foliage was meticulously removed from the stems. The resultant plant material was then organized into bundles and subjected to a drying process within a controlled low-light environment with partial shade for approximately 21 days. After drying, the stem bark was finely ground to a coarse powder using an electric grinder and retained for further use. The produced powder was observed to be notably lightweight.

### 2.3. Organoleptic Evaluation

Sense organs are the most effective and rapid means of assessing the purity of a medicinal plant, thereby ensuring its quality and purity. The organoleptic evaluation of plant powder comprises a systematic assessment using the senses of smell, sight, taste, and touch [8]. This comprehensive evaluation identifies essential quality parameters, including odor, appearance, flavor, texture, and other sensory characteristics pertinent to the powder. Analyzing the powder’s aroma is critical, as deviations in scent may indicate suboptimal storage conditions or the powder’s age, both of which are detrimental to its future utility. Evaluating attributes such as color, texture, and particle size helps determine the visual acceptability of the material, thereby ensuring consistency to mitigate discoloration and contamination risks. The sensory analysis also incorporates tasting, which is vital to discern specific palatability aspects, including bitterness, sweetness, and sourness, inherent to individual plant species [31]. Additionally, the tactile response obtained by rubbing the powder between the fingers enables an assessment of its texture and moisture content, ensuring the powder’s quality for subsequent analyses. It is imperative to confirm that the powder maintains consistent quality across all sensory dimensions—appearance, fragrance, flavor, and texture. Significant discrepancies in these sensory attributes may suggest degradation in quality, contamination, or improper storage practices [31].

### 2.4. Macroscopic Evaluation

Macroscopic analysis focused on the dried stem bark of *A. augustum* (L.) L.f. The plant components were evaluated through direct visual inspection, and a magnifying glass was used to ensure accurate identification [32].

### 2.5. Microscopic Evaluation

A comprehensive analysis of plant cell composition, internal structure, and inclusions can be significantly enhanced by the application of microscopy. This advanced technique facilitates the assessment of the authenticity and purity of medicinal plants by enabling the identification of adulterants and contaminants within herbal formulations. When conducting investigations of plant materials using microscopy, it is crucial to consider essential factors such as the morphology and composition of cellular contents, as well as the size, shape, and relative arrangement of various cells and tissues [33]. In this investigation, thin sections of the stem and stem bark were subjected to staining with Lugol’s and Safranin solutions, followed by microscopic observation and photographic documentation. Anatomical studies are crucial for accurately identifying plant species, thereby facilitating the authentication process of plants.

### 2.6. Powder Microscopic Evaluation

Powder microscopy is a crucial technique in pharmacognosy for identifying and verifying the species of medicinal materials. This method utilizes staining chemicals for quality control and examines the distinct microscopic features of medicinal plants [34]. When plants are processed into powder, their morphological characteristics may not serve as an effective means of identification. In these circumstances, microscopic examination is crucial. Researchers can standardize the microscopic features associated with a particular herbal medicine by analyzing microscopic images [34,35].

In Ayurveda, powdered forms of medicine are among the most widely prescribed options. These powders can contain either a single active compound or a blend of several active compounds. Microscopic examination serves as the most effective method for accurately identifying these substances. Although many plant barks may present similar morphological characteristics after collection and drying, their powdered forms exhibit distinct microscopic features. These unique characteristics are crucial for the precise botanical identification of the materials. The advancement in precision and flexibility in capturing data on microscopic attributes significantly enhances the verification of the authenticity of herbal medications [35].

A small quantity of finely milled stem bark powder was added to a test tube containing a xylene solution, followed by thorough agitation to prepare a sample for analytical examination. Subsequently, several drops of the powdered mixture were deposited onto a microscope slide that had been previously coated with a phloroglucinol solution and allowed to rest for a few minutes. Following this, a few drops of hydrochloric acid (HCl) were applied onto the slide. The resultant slides were then photographed and subjected to microscopic examination.

### 2.7. Histochemical Color Reaction

When treated with chemical reagents, plant samples exhibit specific histochemical color reactions to identify the predominant cellular components [14]. The blue coloration observed as a reaction of iodine with the transverse section (TS) of plant materials, particularly in the spongy parenchyma region, indicates the presence of starch. Treatment of plant parts with iodine and sulfuric acid (H_2_SO_4_) results in a bright yellow color, signifying the presence of cellulose in the chlorenchyma region. The application of safranin for evaluating lignin content results in a red coloration in the vascular zone of the plant components. The presence of mucilage in the spongy parenchyma is evidenced by the solid violet color generated from the interaction of methylene blue with plant tissues. Additionally, the reaction between plant components and amido black at the cambium produces a green tint, demonstrating the presence of proteins [14].

### 2.8. Physicochemical Parameters

All the diverse physicochemical parameters, including total ash content, acid-insoluble ash, water-soluble ash, sulfate ash, foreign matter, loss on drying, foaming index, swelling index, and 10% pH, were systematically quantified [33].

#### 2.8.1. Total Ash Content

Total ash content refers to the inorganic residue that remains following the combustion of plant materials. This residue includes both water-soluble and water-insoluble components of ash.

Total ash (%) = (weight of ash/weight of sample) × 100

#### 2.8.2. Acid-Insoluble Ash

It denotes the residue of ash that remains undissolved when treated with dilute hydrochloric acid, which serves as an indicator of impurities, including silica and other insoluble minerals present in the plant sample.

Acid-insoluble ash (%) = (Weight of acid-insoluble ash/Weight of total ash) × 100

#### 2.8.3. Water-Soluble Ash

It represents the portion of total ash that dissolves in water, reflecting the solubility of inorganic compounds in water. It is utilized to evaluate the quality and purity of a sample.

Water-soluble ash (%) = (Weight of water-soluble ash/Weight of total ash) × 100

#### 2.8.4. Sulfate Ash

It refers to the inorganic residue that remains after a sample undergoes heating in the presence of sulfuric acid, predominantly comprising sulfate ions. This analytical procedure is crucial for detecting sulfate-containing impurities, which may compromise the integrity and quality of the plant sample.

Sulfate ash (%) = Weight of sulfate ash/Weight of total ash) × 100

#### 2.8.5. Foreign Matter

The plant sample is subjected to visual inspection, during which any extraneous materials, including dirt, plant debris, or contaminants, are meticulously removed and quantified by weight.

Foreign matter (%) = (Weight of foreign matter/Total weight of sample) × 100

#### 2.8.6. Loss of Dying

It refers to the quantifiable decrease in the mass of a plant sample subjected to a specified drying temperature, primarily intended to remove moisture content.

Loss on drying (%) = Initial weight − Final weight/Initial weight) × 100

#### 2.8.7. Foaming Index

The Foaming Index evaluates the foaming characteristics of plant-based extracts.

Foaming Index (%) = (Volume of foam/Initial volume of sample) × 100

In this context, the initial sample volume represents the quantity present before agitation, whereas the foam volume denotes the amount produced after shaking. This measurement is typically evaluated at a specified time interval.

#### 2.8.8. Swelling Index

It indicates how well plant materials can take in water and swell, which is essential for drug delivery systems.

Swelling Index (%) = (Weight of swollen sample − Initial volume/Initial volume) × 100

#### 2.8.9. pH of 10% 

The pH value of a 10% *w/v* aqueous solution of the plant sample is vital for evaluating its acidity or alkalinity. This pH level has a significant impact on the stability, solubility, and interactions of the plant material with other materials.

### 2.9. Methanolic Stem Bark Extract Preparation

The methanolic extract of the stem bark, referred to as PME, was obtained using the Soxhlet extraction method, maintaining the temperature between 65 and 70 °C for 72 h. After filtration with Whatman filter paper No. 1, the extract was concentrated using a rotary evaporator maintained at 45 °C. The resulting concentrated methanolic extract was stored in a desiccator for future use.

This study employs methanol as a solvent for preparing plant extracts due to its significant dipole characteristics, which enable the effective dissolution of both polar and nonpolar compounds present in the plant extracts [36]. This results in outcomes that surpass those achieved with other solvents. Additionally, methanol has a low boiling point of 65 °C, which facilitates the efficient removal of the solvent after the reaction. Its cost-effectiveness, broad availability, and widespread use in industrial applications further confirm methanol as a practical choice for extracting plant materials [36,37].

### 2.10. Qualitative Phytochemical Investigation

All qualitative tests were performed according to the pharmacognosy guidelines [38]. Plant materials (5 g each) were placed in five glass containers containing a distinct solvent: methanol, petroleum ether, dichloromethane, benzene, and acetone. The samples were allowed to immerse for seven days at room temperature (25 °C) in dark conditions. To enhance the extraction process, shaking and stirring were applied once daily for seven consecutive days. After the extraction, the mixtures were filtered through Whatman filter paper No. 1, and the resulting concentrates were prepared using a rotary evaporator.

### 2.11. Fluorescence Analysis of Plant Powder with Chemical Reagents

In isolated test tubes, a precise amount of powdered stem bark was systematically subjected to a variety of reagents, including acetic acid, ammonia, iodine, 5% ferric chloride (FeCl_3_), concentrated H_2_SO_4_, concentrated nitric acid (HNO_3_), and 1 N sodium hydroxide (NaOH). After a five-minute reaction, the mixtures were rigorously agitated at ambient temperature and thoroughly examined under both long (365 nm) and short (254 nm) ultraviolet (UV) radiation spectra [8].

### 2.12. Quantification of Total Phenolics and Total Flavonoids

The total phenolic content (TPC) and total flavonoid content (TFC) were estimated using a standard protocol [39]. For TPC, the prepared sample was diluted to a concentration of 1 mg/mL. Then, 1 mL of the extract was aliquoted into test tubes, and each sample was tested in triplicate. Subsequently, 1.5 mL of Folin–Ciocâlteu reagent was added to each test tube. Following this, 1 mL of 7.5% sodium carbonate (Na_2_CO_3_) was added, and the mixture was homogenized using a vortex, resulting in a final volume of 3 mL with methanol. The test tubes were incubated in the light condition at 40 °C for 30 min. The absorbance of the reaction mixture was then measured at 765 nm using a UV–visible spectrophotometer. For TFC analysis, a precisely measured 0.25 mg of the prepared sample was introduced into a test tube, followed by the careful addition of 1 mL of distilled water. All procedures were performed in triplicate to ensure replicability. Subsequently, 750 µL of 5% sodium nitrite (NaNO_3_) was incorporated, and the resultant mixture was homogenized using a vortex mixer to ensure thorough mixing. The test tubes were then incubated in a dark environment for 6 min to minimize exposure to light. Following the incubation period, 150 µL of 5% NaOH and 1 mL of methanol were added to each tube, resulting in a final volume of 3 mL, which was achieved by adding methanol. The absorbance of the solution in all test tubes was then measured at a wavelength of 510 nm using a spectrophotometer.

### 2.13. Antioxidants and Radical Scavenging Activity

PME’s antioxidant and radical scavenging activities were evaluated using standard methodologies, specifically employing DPPH, FRAP, and ABTS assays [39,40,41,42].

### 2.14. In Vitro and Ex Vivo Hepatoprotective Activity

HepG2 cells (procured from the National Centre for Cell Science, Pune, India) were incorporated into the study to assess the hepatoprotective effects of PME at various concentrations (10 µg/mL, 15 µg/mL, and 25 µg/mL). HepG2 cells were cultured in Dulbecco’s Modified Eagle Medium (DMEM), supplemented with 10% phosphate-buffered saline (PBS) and non-essential amino acids, and incubated in a 37 °C environment with 5% carbon dioxide (CO_2_). The impact of different PME concentrations on cell viability was evaluated using the 3-(4,5-dimethylthiazolyl-2)-2,5-diphenyltetrazolium bromide (MTT) assay [43], and absorbance was measured at 570 nm. Additionally, the levels of aspartate aminotransferase (AST), alanine aminotransferase (ALT), and lactate dehydrogenase (LDH) were quantified in supernatants based on the manufacturer’s instructions (Sigma Aldrich, Saint Louis, MO, USA), followed by the colorimetric assay.

Furthermore, to validate the findings, a fresh liver was obtained from a goat sacrificed at a nearby slaughterhouse (National Highway Point, Silchar, India) and preserved in a Krebs–Ringer solution. A sharp knife was used to slice the liver thinly using a Microtome (Digilab India, Delhi, India). These slices were pre-incubated for 60 min in small, plugged beakers containing 10 mL Krebs–Ringer solution, in a shaking water bath, followed by washing with 10 mL Krebs–Ringer solution every 10 min for an hour [44]. Purified liver sections yielded eight distinct culture groups designated for specific treatments. For experimental purposes, 1 mL of 15 mM cytotoxic carbon tetrachloride (CCl_4_), standard treatment silymarin, and PME of *A. augustum* (L.) L.f. were employed [39].

Group I served as a control group, free from toxicants. Group II was administered 1 mL of prepared CCl_4_ solution. Groups III, IV, and V received 1 mL of prepared CCl_4_ and 1 mL of plant extract at varying concentrations of 10, 15, and 25 μg/mL, respectively. Group VI was treated with 1 mL of CCl_4_ (10 μg/mL) and silymarin (10 μg/mL). Groups VII and VIII received 1 mL of PME (25 μg/mL) and silymarin (10 μg/mL). All groups, except for I, VII, and VIII, were incubated with CCl_4_ at 4 °C for 1 h. Following incubation, the treated groups were administered varying concentrations of PME. After the treatment, all cultures underwent a secondary incubation for 2 h at 37 °C in a water bath. Following incubation, the culture media was homogenized with ice-cold normal saline at 4 °C. Biochemical markers, including LPO, ALT, AST, and LDH, were measured by centrifuging each sample, after which a clear supernatant was collected.

## 3. Results

### 3.1. Phytochemical Screening

**Figure 2:**
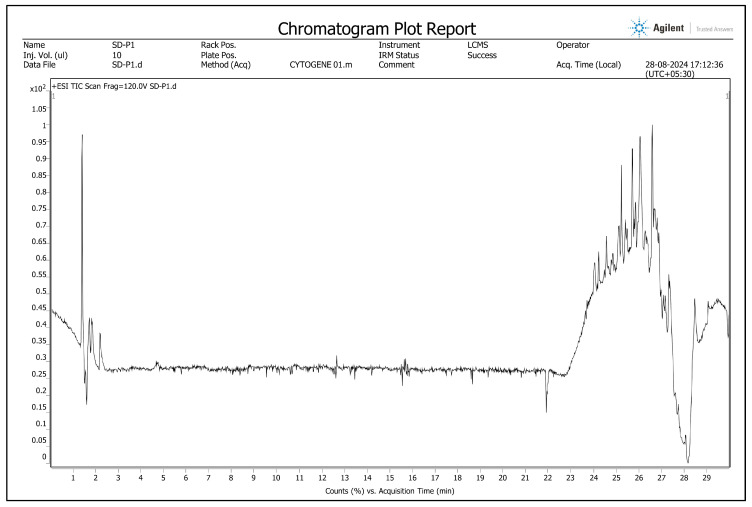
Chromatogram of methanolic stem bark extract of *Abroma augustum* (L.) L.f. generated by liquid chromatography–high-resolution mass spectrometry (LC-HRMS).

A total of fifty (50) phytochemicals were tentatively identified from the stem bark of *A. augustum* (L.) L.f., analyzed using LC-HRMS. They are presented in Table 1, which includes retention time, adduct information, molecular weight, and chemical formula, along with the chromatogram (Figure 2). A total of fifty-five phytochemicals were identified, including eleven alkaloids, ten flavonoids, seven terpenes, eight phenolics, four coumarins, three quinones, one furocoumarin, one benzoic acid, two glycosides, one carboxamidine, one benzofuran, and one adenine derivative.

### 3.2. Pharmacognostic Standardization and Physicochemical Characterization

**Figure 3:**
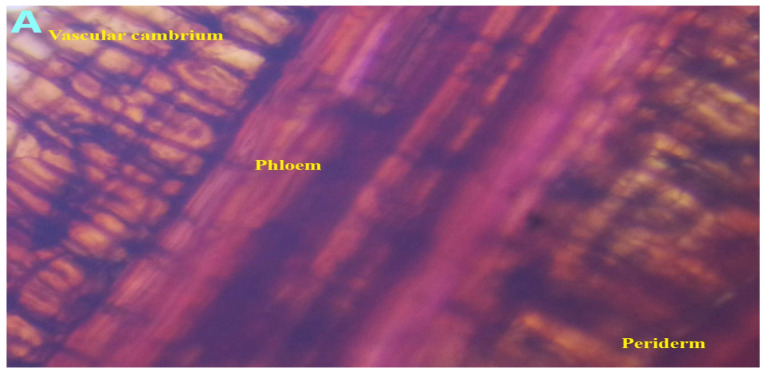

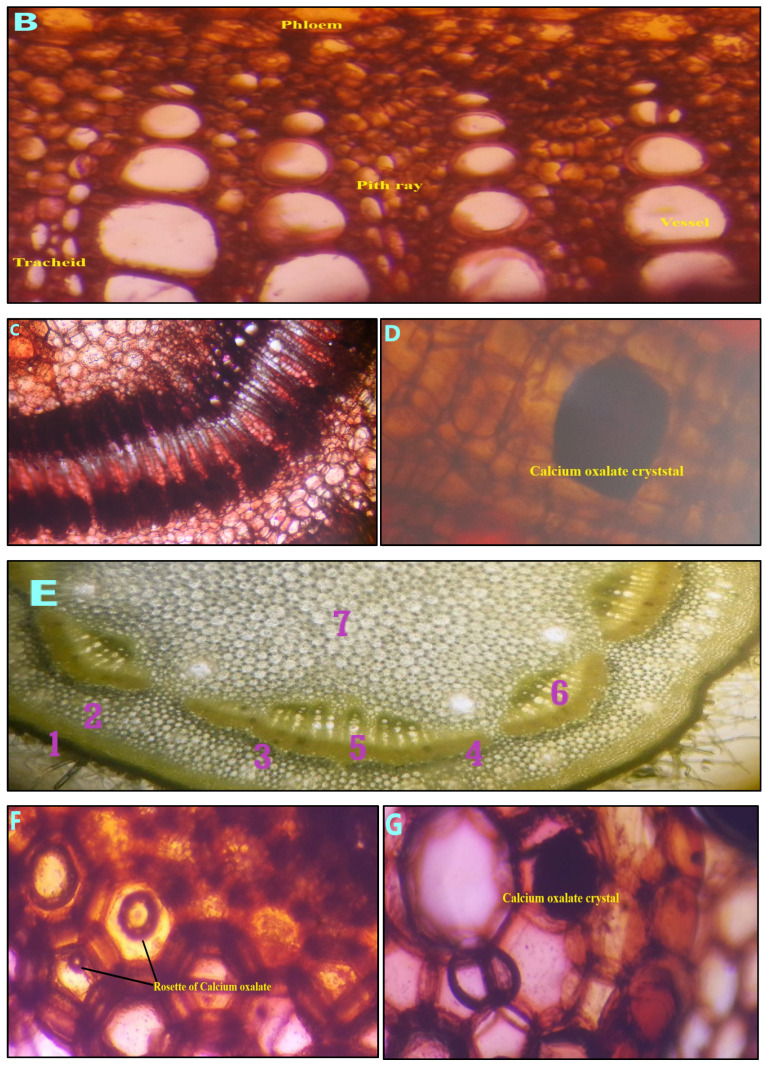
Transverse section of *Abroma augustum* (L.) L.f. stem stained with safranin observed under a microscope (40×). (**A**) Vascular cambium. (**B**) Phloem, vessel, pith rays, and tracheid. (**C**) Deeply stained transverse section. (**D**) Calcium oxalate crystal. (**E**) (1) Epidermis, (2) hypodermis, (3) cortex, (4) sclerenchymatous sheath, (5) phloem, (6) xylem, and (7) pith. (**F**) Calcium oxalate rosette. (**G**) Calcium oxalate.

Organoleptic evaluation of *A. augusta* (L.) L.f. revealed a fresh and mild herbaceous aroma, along with a distinctly bitter taste. The stems displayed coloration varying from light to dark brown, producing an odorless fibrous matrix upon drying. Spiny hairs, linear twigs with branching, and lobed and unlobed leaves identify the plant’s texture. Following staining with safranin and a 5-minute incubation period, thin sections of the plant tissue were examined under a microscope, revealing a cuticle-like structure that enveloped the epidermis. Furthermore, the presence of notable xylem and phloem elements was observed, along with calcium oxalate crystals and rosettes (Figure 3).

**Figure 4:**
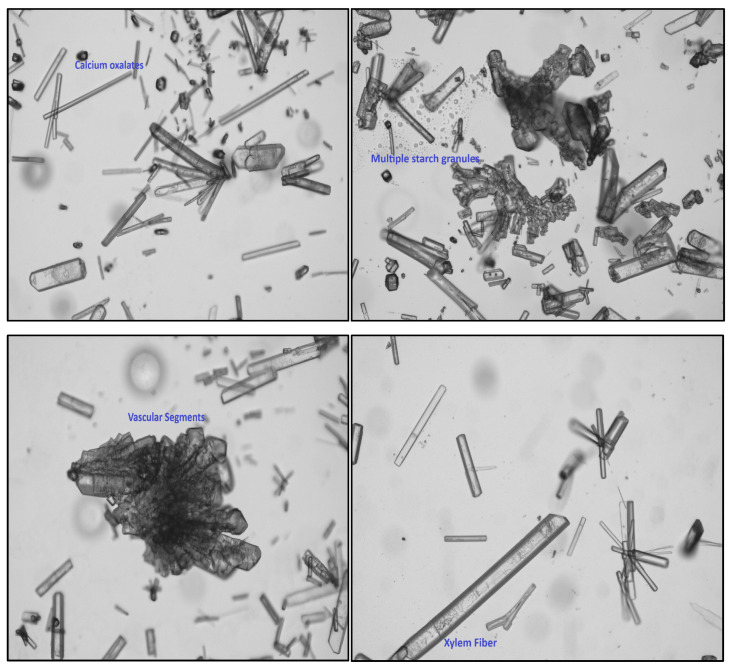
Powder microscopy photographs of xylem fibers, starch grains, vasculature segments, and calcium oxalate.

Microscopic analysis of the powder demonstrated a substantial presence of numerous elongated, narrow-tipped xylem fiber fragments, in addition to distinct starch grains displaying rounded and various other morphological configurations. Vascular components, including segments of both xylem and phloem elements, were also identified (Figure 4).

Calcium oxalate crystals were identified throughout the powdered sample. Physicochemical analysis provided a detailed characterization of the inorganic constituents, potential adulterants, siliceous compounds, and polysaccharides, collectively enhancing our understanding of the plant’s physical and chemical properties. The extract derived from the plant was determined to be suitable for formulating and developing skincare products (Table 2).

### 3.3. Qualitative Phytochemical Investigation and Fluorescence Analysis

A qualitative phytochemical investigation was performed to verify the presence of essential phytoconstituents in the plant bark powder. The extract was prepared utilizing a variety of solvents, encompassing highly polar, moderately polar, and non-polar solvents. The results presented in Table 3 demonstrate that the PME is particularly effective for phytochemical analysis, yielding a high concentration of key phytochemicals, including alkaloids, saponins, phenols, flavonoids, tannins, steroids, and triterpenoids. In comparison with highly polar solvent (methanol), moderately polar solvents (dichloromethane and acetone) and non-polar solvents (petroleum ether and benzene) yielded lower concentrations of phytocompounds present in the plant sample.

Furthermore, the qualitative analysis of the methanolic extract lays the groundwork for subsequent quantitative and pharmacological studies of the plant’s methanolic extracts. Consequently, the methanolic extract of the plant bark was selected for in vitro hepatoprotective investigations, as well as the hot and cold extractive values (Table 4).

Fluorescence analysis of *Abroma augustum* (L.) L.f. stem bark powder treated with various chemical reagents also provided valuable insights into the standardization of the plant powder (Table 5).

### 3.4. Total Phenolic Content (TPC) and Total Flavonoid Content (TFC)

TPC in the PME was quantified using gallic acid as a standard and expressed as mg/g of gallic acid. A calibration curve was established employing the Folin–Ciocâlteu reagent. Various working solutions of gallic acid, with concentrations of 50, 100, 150, and 200 µg/mL, were prepared by diluting a 5 mg/mL stock solution with methanol. Subsequently, 1.5 mL of Folin–Ciocâlteu reagent and 1 mL of Na_2_CO_3_ were added, and the mixture was incubated for 30 min at 40 °C. The absorbance of the resulting colored solution was measured using a UV–visible spectrophotometer at a wavelength of 765 nm. The determined phenolic content of the PME was 12.32 ± 0.01 mg/g of gallic acid.

TFC in the PME was quantified using a quercetin calibration curve, prepared at a concentration of 1 mg/mL in methanol with a total volume of 10 mL. This was diluted with methanol to create working standards at concentrations of 25, 50, 100, and 150 µg/mL. The absorbance for all standard solutions was measured using a UV–visible spectrophotometer at a consistent wavelength of 510 nm. The recorded flavonoid content was determined to be 42.14 ± 3.5 mg/g quercetin.

### 3.5. Quantification of Antioxidant Properties

The antioxidant properties of PME were systematically evaluated using the DPPH assay, which yielded an IC_50_ value of 214.007 µg/mL, indicating moderate antioxidant potential in the methanolic extract. In comparison, the FRAP assay yielded an IC_50_ value of 132.307 µM FeSO_4_ equivalent/mL. Furthermore, the ABTS assay yielded an IC_50_ value of 45.455 µg/mL, providing novel insights into the antioxidant properties of the plant extract. These findings imply that the PME of *A. augustum* (L.) L.f. poses a promising candidate as a natural source of antioxidants. Observed antioxidant properties may be attributed to the Devil’s cotton’s phenolic and flavonoid contents. The present study measured the estimated TPC and TFC at 12.32 ± 0.01 mg/g gallic acid and 42.14 ± 3.5 mg/g quercetin, respectively.

### 3.6. In Vitro Hepatoprotective Activity

The cytotoxic effect of PME on HepG2 cells was investigated, and the results are presented in Table 6.

**Figure 5:**
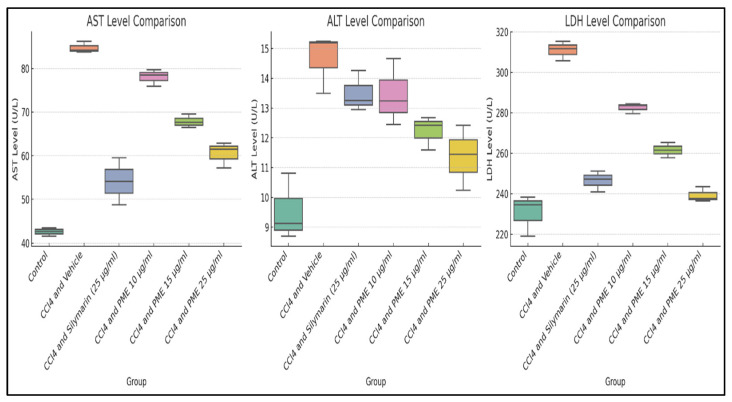
Boxplots for aspartate aminotransferase (AST), alanine aminotransferase (ALT), and lactate dehydrogenase (LDH) levels in HepG2 cells. These provide a visual representation of the variations in AST, ALT, and LDH levels across different experimental groups, highlighting the effects of CCl_4_-induced hepatotoxicity and the ameliorative impact of the methanolic extract of *Abroma augustum* (L.) L.f. stem bark and silymarin.

HepG2 cell supernatants were analyzed for the levels of AST, ALT, and LDH enzymes (U/L) in groups treated with CCl_4_, standard drug silymarin, and various PME concentrations. The results showed that the CCl_4_-treated groups had the highest AST levels (84.70 ± 1.33), indicative of significant hepatotoxicity caused by CCl_4_ treatment, compared to the untreated control (42.56 ± 1.00) and the silymarin group (54.12 ± 5.38). In contrast, the PME treatments (10, 15, and 25 µg/mL) resulted in a dose-dependent decrease in AST levels (78.01 ± 1.94, 67.85 ± 1.55, and 60.50 ± 2.96, respectively), with PME at 25 µg/mL showing the most excellent efficacy. The substantial F statistic (93.18) and *p*-value (<0.0001) confirm the significant differences in AST levels among the experimental groups. The post hoc analysis indicated that PME at a 25 µg/mL dose and silymarin had similar effects, suggesting that PME is a promising hepatoprotective agent (Supplementary File). Regarding ALT levels, the group treated with CCl_4_ showed the highest measurement (14.65 ± 1.00), signifying considerable hepatotoxicity compared to both the untreated control (9.54 ± 1.12) and the silymarin-treated group (13.49 ± 0.69). Administration of PME at varying doses of 10, 15, and 25 µg/mL resulted in a dose-dependent reduction in ALT levels (13.45 ± 1.12, 12.23 ± 0.57, and 11.37 ± 1.09, respectively), with the 25 µg/mL dose demonstrating optimal efficacy. The elevated F statistic (10.87) and *p*-value (<0.0001) further validate the notable differences in ALT levels among the experimental groups. Subsequent post hoc analyses indicated that the 15 and 25 µg/mL doses had enhanced hepatoprotective effects (Supplementary File). In the group treated with CCl_4_, the highest LDH level recorded was 311.03 U/L, indicating severe hepatotoxicity due to CCl_4_ exposure, compared to the untreated control group (230.63 ± 10.31 U/L) and the silymarin group (246.50 ± 5.15 U/L). The application of PME at doses of 10, 15, and 25 µg/mL resulted in a dose-dependent reduction in LDH levels (282.54 ± 2.60, 261 ± 0.375, and 239.26 ± 3.71, respectively), with the lowest LDH value observed at the 25 µg/mL dose. A high F-statistic of 86.20 and a *p*-value of <0.0001 confirm the significant differences in LDH levels among the experimental groups. Post hoc analysis showed that both silymarin and PME at 25 µg/mL doses significantly reduced hepatotoxicity, suggesting a promising hepatoprotective effect against CCl_4_-induced toxicity (Supplementary File). The results for AST, ALT, and LDH enzymes are illustrated in Figure 5.

Within the hepatic system, CCl_4_ undergoes metabolic biotransformation, yielding free radicals that play a pivotal role in LPO and the induction of oxidative stress. These pathological mechanisms result in modifications of hepatic enzyme activities and subsequently precipitate inflammation and apoptotic processes in hepatocytes [44,45]. LPO in hepatocytes occurs due to oxidative damage inflicted on polyunsaturated fatty acids (PUFAs) within cellular membranes. This vulnerability is intrinsically linked to double bonds in PUFAs, rendering them especially susceptible to peroxidation. The initial oxidative event catalyzes a chain reaction that further compromises various lipid components, producing secondary byproducts, including malondialdehyde (MDA) and 4-hydroxy-2-nonenal (4-HNE). These byproducts are known to contribute to additional hepatocellular injury [46]. In the current investigation, the LPO levels were analyzed across diverse experimental groups to determine the antioxidant properties of the PME.

An AST/ALT ratio of 2:1 is considered indicative of hepatic damage. Furthermore, elevated AST levels may suggest comorbidities related to heart, brain, or skeletal-muscle injuries [47]. LDH is an oxidoreductase enzyme that is pivotal in converting pyruvate to lactate and in glucose metabolism under hypoxic conditions. The serum concentration of LDH, indicative of liver-tissue injuries, is, therefore, essential for evaluating liver biochemistry and assessing the overall clinical condition of the liver [48]. In the current investigation, the administration of PME in goat liver homogenate, it was found that CCl_4_ exposure significantly increased the AST level, confirming hepatotoxicity. PME treatment exhibited dose-dependent hepatoprotective effects, particularly at 25 µL/mL, resulting in a notable reduction in AST levels (97.33 ± 13.15) compared to the CCl_4_-treated group (169.00 ± 4.27). In contrast, both silymarin at 10 µg/mL and PME at 25 µg/mL brought the AST level closer to that of the control group (60.66 ± 4.12).

The ALT level increased significantly with CCl_4_ treatment, indicating liver tissue injury. PME at a dose of 25 µg/mL showed a substantial decrease in ALT levels (165.67 ± 3.52), approaching that of the silymarin-treated group (85.67 ± 53.35). Silymarin alone at a dose level of 10 µg/mL exhibited the most significant hepatoprotective effect (43.75 ± 5.38). Regarding LDH, PME at 15 and 25 µg/mL doses showed the most promising results, with 25 µg/mL highlighting a substantial protective effect (23.33 ± 1.86). Silymarin alone at 10 µg/mL (7.75 ± 0.40) had the most notable hepatoprotective impact, reducing LDH levels closer to the control (12.33 ± 2.53).

**Figure 6:**
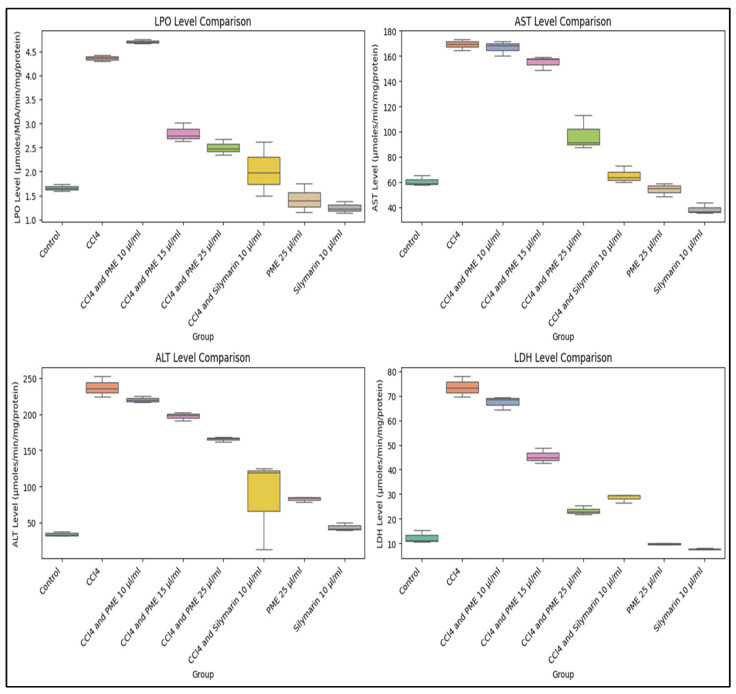
Effect of stem bark extract of *Abroma augustum* (L.) L.f. and silymarin on hepatic biomarkers—lipid peroxidation (LPO), aspartate aminotransferase (AST), alanine aminotransferase (ALT), and lactate dehydrogenase in CCl_4_-treated goat liver homogenates. The figure presents boxplots illustrating the distribution of levels across different experimental groups.

This study indicated that CCl_4_ significantly induced hepatotoxicity, as evidenced by the increased levels of LPO, AST, ALT, and LDH. PME at a 25 µg/mL dose showed the highest hepatoprotective effect (Figure 6).

On the other hand, while silymarin remains the most effective, PME at 25 µg/mL demonstrated comparable protective effects, particularly in reducing AST, ALT, and LDH.

The ANOVA results (Table 7) indicate the significant difference among the LPO, AST, ALT, and LDH treatment groups. Post hoc analysis revealed that CCl_4_ significantly increased the parameters, as mentioned earlier, compared to the control (*p* < 0.0001). The PME at 25 µg/mL is more effective than the PME at lower doses (10 and 15 µL/mL); however, it is not as effective as silymarin. Regarding ALT and LDH levels, PME at 25 µg/mL and silymarin at 10 µg/mL show non-significant differences (*p* > 0.05), indicating that both treatments may have similar hepatoprotective effects. And LDH and ALT levels in PME 25 µg/mL are not significantly different from the untreated control group (Supplementary File), indicating that this dose could offer protection against liver injury.

## 4. Discussion

The findings regarding phytochemicals are highly significant, as the phytochemical profiling aspects of the plant are only partially understood. The presence of alkaloids, flavonoids, phenolics, and terpenes identifies a potential chemical profile of the plant, providing preliminary evidence that supports its possible pharmaceutical properties. Significant alkaloids, such as protopine [49], berberine [50], and stachydrine [51]; flavonoids, including sideroxylin [52], morin [53], and galangin [54]; and terpenes like kahweol [55] yield valuable insights into the plant’s pharmaceutical efficacy in addressing a variety of ailments, including pain management, infectious diseases, cancer, cardiovascular illness, and inflammatory conditions. The synergistic interactions of these compounds may lead to innovative approaches in drug development, facilitating the discovery of new therapeutic agents that provide enhanced efficacy in treatment.

Standardizing *A. augustum* (L.) L.f. provides critical insights into its pharmacognostic and physicochemical properties, thus underscoring its suitability for incorporation into herbal formulations and drug development. Morphological and anatomical analyses reveal the presence of a cuticle lining on the epidermis, as well as distinct xylem and phloem structures that feature calcium oxalate crystals and rosettes. This information is instrumental in differentiating *A. augustum* (L.) L.f. from other closely related plant species, serving as a potential indicator of quality assurance. Moreover, analyses revealed a low percentage of total ash, acid-soluble ash, water-soluble ash, and reduced sulfate ash, suggesting minimal inorganic content, fewer adulterants, and high purity in the plant methanolic extract. The higher concentrations of phytoconstituents, including alkaloids, phenolics, flavonoids, tannins, and terpenes, further substantiate the plant’s potential for developing effective herbal formulations [8,56]. *A. augustum* (L.) L.f. may enhance the therapeutic efficacy of herbal formulations, as it has a high swelling index (100%), indicating high hydrophilicity of the plant extract and, thus, suggesting excellent solubility and bioavailability, potentially aiding in the rapid release of bioactive compounds to the target area. The high swelling index is particularly noteworthy, as it suggests that the plant extract can be absorbed more efficiently by the body, potentially resulting in quicker and more effective therapeutic outcomes. Additionally, a foaming index exceeding 100 signifies a substantial concentration of saponins within the extract, which enhances its emulsification properties and provides the herbal formulation with remarkable stability and suitability for topical application [14,51].

The qualitative phytochemical investigation conclusively established that PME is rich in a diverse array of secondary metabolites, including alkaloids, saponins, phenolics, flavonoids, tannins, steroids, and triterpenes. Each assay conducted on the corresponding phytochemical groups yielded positive results, affirming the presence of these secondary metabolites with notable sensitivity. In stark contrast, secondary metabolites were also detected in both moderately polar solvents (dichloromethane and acetone) and non-polar solvents (petroleum ether and benzene), albeit at reduced concentrations, as several assays for the respective phytochemical groups produced negative responses. This indicates the limited extraction efficacy of phytochemicals from the plant sample by these solvents, suggesting their ineffectiveness in isolating the desired phytocompounds. This finding indicates that a polar methanolic solvent demonstrates superior efficacy in extracting phytocompounds from plants compared to less polar and/or non-polar solvents. This enhanced performance is likely due to methanol’s greater affinity for the hydrophilic compounds present in plant extracts, including alkaloids, phenolics, and glycosides. Furthermore, methanol’s ability to penetrate plant membranes is more pronounced than other solvents, facilitating optimal elution of phytocompounds from the plant materials [37,57,58]. Like our work, Truong and his team (2019) found that methanol is an ideal solvent for extracting the maximum number of secondary metabolites from plants. They reported methanol as the most effective solvent for obtaining the maximum yield of phenolics and flavonoids [59].

In addition, the fluorescence of the plant powder was systematically evaluated in this study under visible light, as well as at wavelengths of 254 nm and 365 nm, in the presence of various chemical agents to standardize the plant powder. This assessment of the fluorescent characteristics of the plant may further contribute to quality-assurance protocols.

Phenolics represent a diverse group of plant secondary metabolites synthesized through the shikimate pathway. These compounds play critical physiological roles in plants, including defense mechanisms against pathogens, herbivores, and parasitic invasions, as well as protection from ultraviolet radiation. Additionally, phenolics contribute to the coloration of flowers and are widely distributed throughout various plant tissues. Over eight thousand distinct phenolic structures have been identified, ranging from simple phenolic acids to more complex tannins [60]. Conversely, plant flavonoids represent a subclass of phenolic compounds that play a crucial role in regulating plant cell growth, facilitating pollination, and mitigating both biotic and abiotic stress [61]. The antioxidant properties of plant phenolics and flavonoids have been studied extensively over the past few years. Phenolic compounds enhance plant antioxidant properties through mechanisms such as hydrogen atom donation, single electron transfer, chelation of metal ions, and sequential proton loss coupled with electron transfer [62]. In contrast, plant flavonoids can quench free radicals and chelate metal ions, contributing to the PME’s antioxidant properties [63]. Thus, substantial amounts of phenolics and flavonoids synergistically contribute to the antioxidant properties of the PME [35]. They are found to be effective against an array of ROS and RNS through targeting and neutralizing singlet oxygen (^1^O_2_) [64], superoxide anion (O_2_^−^) [65], hydroxyl radical (•OH), peroxyl radical (ROO•) [66], nitric oxide (NO•) [67], nitrite (NO_2_^−^) [68], nitrate (NO_3_^−^), peroxynitrite (ONOO^−^), and nitrosyl radical (NO^2^•) [69,70], thereby protecting against cellular oxidative damage. Thus, the plant’s antioxidant potential directly relates to its abundance of phenolic and flavonoid content [71]. Additionally, plants with antioxidant properties are often associated with hepatoprotective activity [43,72]. Based on the TPC (12.32 ± 0.01 mg/g gallic acid) and TFC (42.14 ± 3.5 mg/g quercetin) contents of PME, as noted in the present study, it may be hypothesized that the plant *A. augustum* (L.) L.f. used by the Tripuri community as a traditional medicine has antioxidant potential and may be a promising candidate hepatoprotectant. This study evaluated the antioxidant mechanism of PME and its hepatoprotective effect on CCl_4_-induced hepatotoxicity in goat liver homogenate and HepG2 cells.

Free radical scavenging activity of *A. augustum* (L.) L.f. bark methanolic extract was evaluated using DPPH, FRAP, and ABTS assays, and the results were expressed as IC_50_. The IC_50_ values of DPPH, FRAP, and ABTS were recorded as 214.007 µg/mL, 132.307 µg/mL, and 45.455 µg/mL, respectively.

The DPPH assay results for PME indicate a moderate antioxidant potential in scavenging stable DPPH radicals. However, it is essential to note that this preliminary test predominantly reflects PME’s capacity to scavenge free radicals. The discrepancies observed between the DPPH assay and the results from the other two assays may be attributed to the comparatively larger size of the DPPH radicals, which could impede PME’s efficacy in scavenging free radicals and potentially result in inaccurate assessments [73]. On the other hand, the ABTS assay stands out as the most sensitive among the three assays employed in the present study. The heightened sensitivity, attributed to ABTS’s unique method of operation, instills confidence in the reliability of our results. By measuring the capacity of antioxidants to scavenge the ABTS•+ radical in an aqueous solution, the ABTS assay can detect a wide range of antioxidants, including hydrophilic and lipophilic compounds [74,75]. In comparison, the FRAP assay measures the ability of antioxidants to reduce ferric ions (Fe^3+^) to ferrous ions (Fe^2+^) only [75,76].

The variability in assay results among the three methodologies suggests that the methanolic bark extract may exhibit distinct scavenging mechanisms depending on the radical species involved. Consequently, the present study highlights the potential of PME as an antioxidant; however, further characterization employing more sensitive assays is necessary to optimize its antioxidant capabilities for clinical applications fully.

CCl_4_-induced hepatotoxicity is a widely recognized and reproducible experimental model used to investigate the hepatoprotective effects of various plant extracts [45,77,78]. CCl_4_ treatment significantly increased the LPO level in goat liver homogenate (4.36 ± 0.067), indicating the presence of oxidative stress and lipid peroxidation serving as a standard reference for CCl_4_-induced hepatotoxicity. High levels of LPO indicate cellular oxidative damage to living cells upon CCl_4_ insult [44]. The PME treatment at a dose of 10 µg/mL did not reduce LPO, whereas doses of 15 and 25 µg/mL significantly decreased LPO levels (2.80 ± 0.19 and 2.50 ± 0.16, respectively). Silymarin at 10 µg/mL and PME alone at 25 µg/mL showed promising results by reducing CCl_4_-induced lipid peroxidation (LPO). The promising results of PME at dose levels of 15 and 25 µg/mL indicate a protective effect against cellular oxidative stress through the reduction of LPO.

Several studies have confirmed that the polyphenols in plant extracts exert a modulatory effect on LPO, possibly due to their redox properties, conjugate rings, and carboxyl groups [79,80]. In this study, the dose-dependent reduction of LPO levels suggested that TPC and TFC synergistically work through multiple modes of action, including free radical scavenging, prevention of free radical production, hydrogen donation, chelation of metal ions [72], and modulation of enzymatic antioxidant action [81].

The liver plays a crucial role in digestion, metabolism, and detoxification of numerous bodily substances. Biochemical markers, including enzymes such as ALT, AST, and LDH, are commonly utilized to evaluate liver function. An elevation in these critical enzymes is indicative of hepatocellular damage [82]. ALT is primarily involved in converting L-alanine to α-ketoglutarate, thereby facilitating the production of L-glutamate and pyruvate, in turn aiding in cellular energy production. Increased ALT levels have been linked to liver injury, jaundice, infection, inflammation, hepatitis, and cirrhosis [83,84]. AST is another significant biochemical marker often evaluated alongside ALT. A comparative analysis of both marker aids in the clinical diagnosis of liver conditions [82]. In this study, we evaluated the hepatoprotective effect of the plant extract by assessing the modulation of three key liver enzyme biomarkers (AST, ALT, and LDH), which indicate liver function and cellular integrity. The significance of these three liver enzymes in evaluating the hepatoprotective effect of the plant extract has been established in earlier studies [44,85,86]. The findings suggested that significant restoration of AST levels in HepG2 cells and goat liver homogenate at doses of 15 and 25 µg/mL holds the promising hepatoprotective effect of PME. Likewise, a significant restoration was observed in a dose-dependent manner (10, 15, and 25 µL/mL) for ALT and LDH levels in both HepG2 cells and goat liver homogenate against CCl_4_-induced hepatotoxicity. Thus, the findings of this study suggest dose-dependent restoration of liver enzymes, showing the presence of promising hepatoprotectants and antioxidants in the PME. CCl_4_ insult increased the concentration of trichloromethyl radicals (CCl^3+)^ in liver cells and produced toxic intermediates through the reductive metabolism of CCl^3+^. These intermediates initiated the leakage of serum enzymes, leading to the peroxidation of membrane lipids, depletion of antioxidants, and ultimately triggering necrosis [86]. In goat liver and HepG2 cells, PME restored AST, ALT, and LDH levels, as previously reported [21,44]. The findings of the present study concur with their conclusions. It may thus be speculated that the hepatoprotective action of *A. augustum* (L.) L.f. methanolic extract might be due to several mechanisms. PME may potentially mitigate CCl_4_-induced toxicity by redox-balancing in liver cells, increasing antioxidant levels, blocking inflammatory mediators, directly protecting against hepatotoxins, promoting lipid homeostasis, and inhibiting inflammatory mediators [87,88,89,90].

Based on the biochemical analyses and statistical results, this study successfully demonstrates the ameliorative potential of *Abroma augustum* (L.) L.f. PME against CCl_4_-induced hepatotoxicity. The dose-dependent reduction in oxidative stress and enzyme levels suggests that PME can effectively protect liver cells from damage. Based on this, we can consider the *Abroma augustum* (L.) L.f. as a potential therapeutic agent for liver toxicity. The mechanism of action is likely to involve antioxidant activity, membrane stabilization, and enzymatic regulation. Further research is essential to clarify the hepatoprotective mechanisms of PME. This involves histopathological examination, exploring additional biochemical parameters following PME treatment, and analyzing molecular pathways, with a focus on key signaling pathways that highlight the hepatoprotective effects of PME.

## 5. Conclusions

The present study provides a comprehensive understanding of *A. augustum* (L.) L.f. through phytochemical screening, and pharmacognostic and physicochemical validation. The presence of notable alkaloids like protopine, berberine, and stachydrine; flavonoids such as sideroxylin, morin, and galangin; and terpenes, including kahweol, along with other significant phytochemicals from the plant’s bark, as highlighted by LC-HRMS analysis, underscores the chemical diversity found in the plant and broadens our understanding of its phytochemical profile. It is significant in reporting, possibly for the first time, the antioxidant properties and hepatoprotective effects of PME. High levels of phenolic and flavonoid compounds are believed to contribute to the antioxidant and hepatoprotective activities of Devil’s cotton. An in vitro study utilizing HepG2 cells and an ex vivo study using goat liver homogenate have demonstrated that the methanolic extract of the plant has the potential to mitigate oxidative stress and protect against liver damage. Consequently, these extracts may contribute to the formulation of hepatoprotective pharmaceuticals, owing to their abundance in polyphenolic compounds. However, further research utilizing animal models is strongly recommended to comprehensively evaluate the therapeutic efficacy and safety profile of the plant extract, as well as to assess and mitigate any potential adverse toxic effects.

## Figures and Tables

**Table 1:** Phytochemicals from *Abroma augustum* (L.) L.f. by liquid chromatography–high-resolution mass spectrometry (LC-HRMS).

Sl. No.	Name of the Compound	Possible Nature	Retention Time (minutes)	Adduct	Molecular Weight (g/mol)	Chemical Formula
1	Protopine	Alkaloids	25.99932	[M + NH_4_]^+^	353.4	C_20_H_19_NO_5_
2	Stachydrine		1.8074	[M + H]^+^	143.18	C_7_H_13_NO_2_
3	Tsitsikammamine A		25.86585	[M + H]^+^	303.3	C_18_H_13_N_3_O_2_
4	3-methyloxindole(oxindoles)		24.51443	[M + H]^+^	313.3	C_9_H_9_NO
5	Heliamine		26.68337	[M + H]^+^	193.24	C_11_H_15_NO_2_
6	(−)-caaverine		25.41538	[M + H]^+^	267.32	C_17_H_17_NO_2_
7	Berberine		24.78138	[M + Na]^+^	336.4	C_20_H_18_NO_4_^+^
8	Caffeine		24.7647	[M + H]^+^	194.19	C_8_H_10_N_4_O_2_
9	Harmine		24.23082	[M + H]^+^	212.25	C_13_H_12_N_2_O
10	Theobromine		25.24853	[M + H]^+^	180.16	C_7_H_8_N_4_O_2_
11	9-methoxycanthin-6-one		25.7157	[M + H]^+^	250.25	C_15_H_10_N_2_O_2_
12	Sideroxylin	Flavonoids	27.1672	[M + H]^+^	312.3	C_18_H_16_O_5_
13	Acacetin		27.65103	[M + Na]^+^	284.26	C_16_H_12_O_5_
14	Galangin		28.56867	[M + H]^+^	270.24	C_15_H_10_O_5_
15	Morin		1.723983	[M + K]^+^	302.23	C_15_H_10_O_7_
16	Daidzein		1.406983	[M + H]^+^	254.24	C_15_H_10_O_4_
17	Tectochrysin		24.54782	[M + H]^+^	268.26	C_16_H_12_O_4_
18	6-desmethylsideroxylin		27.1672	[M + H]^+^	298.29	C_17_H_14_O_5_
19	Pinocembrin		26.11612	[M + K]_+_	256.25	C_15_H_12_O_4_
20	Hesperetin		25.23185	[M + K]^+^	302.28	C_16_H_14_O_6_
21	Sanggenon G		27.1672	[M + H]^+^	694.7	C_40_H_38_O_11_
22	Kahweol	Terpenes	1.8074	[M + Na]^+^	314.4	C_20_H_26_O_3_
23	Dihydroactinidiolide, (+/−)		26.016	[M + H]^+^	180.24	C_11_H_16_O_2_
24	8-deoxylactucin		26.36637	[M + H]^+^	438.2	C_15_H_16_O_4_
25	Furanoheliangolide		25.94927	[M + H]^+^	360.4	C_19_H_20_O_7_
26	Nakijiquinone H		27.71778	[M + H]^+^	456.6	C_26_H_40_N_4_O_3_
27	Loliolide		24.58118	[M + H]^+^	196.24	C_11_H_16_O_3_
28	12b-O-[deca-2E,4Z-dienoyl]-13a-isobutyl-4b-phorbol		28.10152	[2M + Na]^+^	584.7	C_34_H_48_O_8_
29	Selaginpulvilin M	Phenolics	27.65103	[M + Na]^+^	524.6	C_36_H_28_O_4_
30	3’,4’,5’-trimethoxyacetophenone		24.54782	[M + H]^+^	210.23	C_11_H_14_O_4_
31	(2-Hydroxy-5-methoxyphenyl) (4-hydroxyphenyl)methanone		26.71673	[M + Na]^+^	244.24	C_14_H_12_O_4_
32	2-acyl-4,6-bisprenylphloroglucinol		27.2673	[M + Na]^+^	290.4	C_17_H_22_O_4_
33	Chlorogenic acid		26.61663	[M + H]^+^	354.31	C_16_H_18_O_9_
34	3-(8-hydroxyoctyl)phenol		26.86688	[M + H]^+^	222.32	C_14_H_22_O_2_
35	Resveratrol		25.79912	[M + H]^+^	228.24	C_14_H_12_O_3_
36	Cornexistin		23.98055	[M + H]^+^	308.33	C_16_H_20_O_6_
37	Demethoxycurcumin	Coumarins	26.8502	[M + H]^+^	338.4	C_20_H_18_O_5_
38	Scopoletin		12.18487	[M + Na]^+^	192.17	C_10_H_8_O_4_
39	Umbelliferone		1.723983	[M + H]^+^	162.14	C_9_H_6_O_3_
40	Urolithin A		1.406983	[M + H]^+^	228.20	C_13_H_8_O_4_
41	Questin	Quinones	26.16617	[M + H]^+^	284.26	C_16_H_12_O_5_
42	2-methoxy-1,4-naphthoquinone		24.0306	[M + H]^+^	188.18	C_11_H_8_O_3_
43	2-Hydroxy-3-(3-methylbut-2-enyl)-1,4-naphthoquinone		24.0306	[M + H]^+^	242.27	C_15_H_14_O_3_
44	Oxypeucedanin	Furocoumarin	25.98263	[M + H]^+^	286.28	C_16_H_14_O_5_
45	2,4,5-rimethoxybenzoic acid	Benzoic acid	25.24853	[M + H]^+^	212.20	C_10_H_12_O_5_
46	Verbascoside	Glycosides	26.71673	[2M + Na]^+^	624.6	C_29_H_36_O_15_
47	Gentiopicrin		27.15052	[M + Na]^+^	356.32	C_16_H_20_O_9_
48	Ectoine	Carboxamidine	1.406983	[M + 2H]^2+^	142.16	C_6_H_10_N_2_O_2_
49	Licarin A	Benzofuran	26.016	[M + Na]^+^	326.4	C_20_H_22_O_4_
50	3-Methyladenine	Adenine derivative	1.8074	[M + H]^+^	149.15	C_6_H_7_N_5_

**Table 2:** Physicochemical analysis of stem bark of *Abroma augustum* (L) L.f.

Physicochemical Parameters (%) Except 10% pH	Obtained Values (%) Except 10% pH
Total ash	6.00
Acid insoluble ash	1.18
Water soluble ash	1.40
Sulphated ash	2.1
Foreign matter	0.00
Loss on drying	1.46
Foaming index	>100
Swelling index	100
10% pH	6.8

**Table 3:** Qualitative phytochemical screening of *Abroma augustum* (L.) L.f. extract.

Phytochemical	Reagent/Test	Methanolic Extract	Petroleum Ether	Dichloromethane	Benzene	Acetone
Alkaloids	Dragendorff’s	+	+	+	+	+
	Mayer’s	+	+	−	+	−
	Hager’s	+	+	+	+	−
	Wagner’s	+	+	−	+	−
Saponins	Foam	+	+	+	+	+
Phenols	Ferric chloride	+	+	−	+	+
	Lead acetate	+	+	+	+	−
	Gelatin	+	−	+	+	−
	Mayer’s	+	+	−	+	+
Flavonoids	Shinnoda’s	+	+	−	+	+
	Lead acetate	+	+	+	+	−
	Alkaline reagent	+	+	+	+	−
Tannins	Gelatin’s	+	+	+	+	−
Steroids and triterpenoids	Salkowski	+	+	+	+	+
	Libermann–Burchard	+	+	−	+	+

**Table 4:** Extractive values of *Abroma augustum* (L.) L.f. in various solvents.

Plant Part	Solvent	Cold Extraction	Hot Extraction
Stem bark	Methanol	8	8.6
	Petroleum ether	7.4	7.9
	Dichloromethane	6.8	7.2
	Benzene	6.7	7.4
	Acetone	7	6.9

**Table 5:** Fluorescence analysis of stem bark powder of *Abroma augustum* (L.). L.f.

Chemicals Used	Visible Light	Short UV (254 nm)	Long UV (365 nm)
1 N NaOH (aq.)	Light yellowish-green	Light green	Purple
1 N NaOH (alc.)	Light pink	Light green	Dark blue
1 N HCl	Pale cream	Pale green	Blue
H_2_SO_4_ (1:1)	Brown	Light green	Black
HNO_3_ (1:1)	Light yellow	Light green	Black
NH_3_	Light greenish yellow	Light green	Blue
Iodine	Bright yellow	Bright green	Black
5% FeCl_3_	Bright yellow	Bright green	Black
Acetic acid	Pale green	Light green	Blue

**Table 6:** Effect of *Abroma augustum* (L.) L.f. stem bark methanolic extract on the viability of HepG2 cells.

Culture Condition.	Concentration (µg/mL)	Cell Viability (%)
Vehicle (PBS)	-	97.66 ± 0.33
Silymarin	10 µg/mL	94.66 ± 0.33 *
PME	10 µg/mL	92.66 ± 1.20 *
PME	15 µg/mL	91.00 ± 0.57 **
PME	25 µg/mL	87.66 ± 0.33 **

Note: After incubation for 24 h, an MTT assay was performed, and the results were expressed as mean ± SD (** considered for significance of results at *p* < 0.001, and * for significant results at *p* < 0.05). A post hoc Tukey test was performed after one-way ANOVA.

**Table 7:** The analysis of variance (ANOVA) test was conducted for lipid peroxidation (LPO), aspartate aminotransferase (AST), alanine aminotransferase (ALT), and lactate dehydrogenase levels, and the results indicate significant differences among the groups for all parameters.

Parameter	Sum of Squares (Between Groups)	df	F-Value	*p*-Value
LPO	35.87	7	81.42	2.58 × 10^−11^
AST	62,678.20	7	189.09	3.58 × 10^−14^
ALT	138,834.63	7	36.47	1.14 × 10^−8^
LDH	14,120.11	7	327.95	4.61 × 10^−16^

## Data Availability

The original contributions presented in this study are included in the article. Further inquiries can be directed at the corresponding author(s).

## Supplementary Materials

The following supporting information can be downloaded at: https://www.mdpi.com/article/10.3390/antiox14040472/s1, Table S1: AST Levels (U/L) in Different Experimental Groups; Table S2: Tukey HSD Post-Hoc Analysis for AST Levels; Table S3: ALT Levels (U/L) in Different Experimental Groups; Table S4: Tukey HSD Post-Hoc Analysis for ALT Levels; Table S5: LDH Levels (U/L) in Different Experimental Groups; Table S6: Tukey HSD Post-Hoc Analysis for LDH Levels; Table S7: LPO Level (µmoles/MDA/min/mg protein) in Goat liver homogenate; Table S8: AST Level (µmoles/min/mg protein) in goat liver homogenate; Table S9: ALT Level (µmoles/min/mg protein) in goat liver homogenate; Table S10: LDH Level (µmoles/min/mg protein) in goat liver homogenate; Table S11: Tukey HSD Post-Hoc Analysis for LPO Levels; Table S12: Tukey HSD Post-Hoc Analysis for AST Levels; Table S13: Tukey HSD Post-Hoc Analysis for AST Levels; Table S14: Tukey HSD Post-Hoc Analysis for ALT Levels; Table S15: Tukey HSD Post-Hoc Analysis for LDH Levels.

## Abbreviations

4-HNE	4-hydroxy-2-nonenal
ABTS	2,2′-azino-bis-(3-ethylbenzothiazoline-6-sulfonic) acid
ALT	Alanine aminotransferase
AST	Aspartate aminotransferase
CCl_3_^+^	Trichloromethyl radicals
CCl_4_	Carbon tetrachloride
CO_2_	Carbon dioxide
DMEM	Dulbecco’s Modified Eagle’s Medium
DPPH	2,2-diphenyl-1-picrylhydrazyl
Fe^2+^	Ferrous ions
Fe^3+^	Ferric ions
FeCl_3_	Ferric chloride
FRAP	Ferric-reducing antioxidant power
HepG2	Human hepatocellular carcinoma G2
H_2_SO_4_	Sulfuric acid
HNO_3_	Nitric acid
IC_50_	Inhibitory concentration at 50%
LC-HRMS	Liquid chromatography–high-resolution mass spectrometry
LDH	Lactate dehydrogenase
LPO	Lipid peroxidation
MDA	Malondialdehyde
MTT	3-(4,5-dimethylthiazolyl-2)-2,5-diphenyltetrazolium bromide
Na_2_CO_3_	Sodium carbonate
NaOH	Sodium hydroxide
PBS	Phosphate-buffered saline
PME	Plant methanolic extract
PUFA	Polyunsaturated fatty acid
RNS	Reactive nitrogen species
ROS	Reactive oxygen species
TFC	Total flavonoid content
TPC	Total phenolic content
TS	Transverse section
WHO	World Health Organization
