# Supplementary material for: Phytochemical Screening, Pharmacognostic Characterization, Antioxidant Activity, and Hepatoprotective Effects of Abroma augustum (L.) L.f. on Human Hepatocellular Carcinoma (HepG2) Cells and Goat Liver Homogenate
Source: Antioxidants (Basel). 2025 Apr 15;14(4):472. doi: 10.3390/antiox14040472 (PMC12024031; doi:10.3390/antiox14040472)
Supplement: Supplementary file 1 [file antioxidants-14-00472-s001.zip › antioxidants-3540928-supplementary.pdf]

Table S1: Aspartate aminotransferase (AST) levels (U/L) in HepG2 cells.

Group	Mean	Standard Deviation (SD)
Control	42.56	1.00
CCl_4 and Vehicle	84.70	1.33
CCl_4 and Silymarin (25 µg/ml)	54.12	5.38
CCl_4 and PME (10 µg/ml)	78.01	1.94
CCl_4 and PME (15 µg/ml)	67.85	1.55
CCl_4 and PME (25 µg/ml)	60.50	2.96

ANOVA results for aspartate aminotransferase (AST) levels (U/L): F-statistic = 93.18, p-value = 0.0000

Table S2: Tukey's Honestly Significant Difference (HSD) post-hoc analysis for aspartate aminotransferase (AST) levels (U/L) in HepG2 cells.

Group 1	Group 2	Mean Difference	p-adj	Lower Bound	Upper Bound	Significant (Reject H_0)
CCl_4 and PME 10 µg/ml	CCl_4 and PME 15 µg/ml	-10.16	0.0078	-17.805	-2.515	Yes
CCl_4 and PME 10 µg/ml	CCl_4 and PME 25 µg/ml	-17.51	0.0001	-25.155	-9.865	Yes
CCl_4 and PME 10 µg/ml	CCl_4 and Silymarin (25 µg/ml)	-23.89	0.0000	-31.535	-16.245	Yes
CCl_4 and PME 10 µg/ml	CCl_4 and Vehicle	6.69	0.0999	-0.955	14.335	No
CCl_4 and PME 10 µg/ml	Control	-35.45	0.0000	-43.095	-27.805	Yes
CCl_4 and PME 15 µg/ml	CCl_4 and PME 25 µg/ml	-7.35	0.0621	-14.995	0.295	No

CCl_4 and PME 15 μg/ml	CCl_4 and Silymarin (25 μg/ml)	-13.73	0.0006	-21.375	-6.085	Yes
CCl_4 and PME 15 μg/ml	CCl_4 and Vehicle	16.85	0.0001	9.205	24.495	Yes
CCl_4 and PME 15 μg/ml	Control	-25.29	0.0000	-32.935	-17.645	Yes
CCl_4 and PME 25 μg/ml	CCl_4 and Silymarin (25 μg/ml)	-6.38	0.1243	-14.025	1.265	No
CCl_4 and PME 25 μg/ml	CCl_4 and Vehicle	24.2	0.0000	16.555	31.845	Yes
CCl_4 and PME 25 μg/ml	Control	-17.94	0.0001	-25.585	-10.295	Yes
CCl_4 and Silymarin (25 μg/ml)	CCl_4 and Vehicle	30.58	0.0000	22.935	38.225	Yes
CCl_4 and Silymarin (25 μg/ml)	Control	-11.56	0.0028	-19.205	-3.915	Yes
CCl_4 and Vehicle	Control	-42.14	0.0000	-49.785	-34.495	Yes

Table S3: Alanine aminotraferase (ALT) levels (U/L) in HepG2 cells.

Group	Mean	Standard Deviation (SD)
Control	9.54	1.12
CCl_4 and Vehicle	14.65	1.00
CCl_4 and Silymarin (25 μg/ml)	13.49	0.69
CCl_4 and PME (10 μg/ml)	13.45	1.12
CCl_4 and PME (15 μg/ml)	12.23	0.57
CCl_4 and PME (25 μg/ml)	11.37	1.09

ANOVA results for alanine aminotraferase (ALT) levels (U/L): F-statistic = 10.87, p-value = 0.0004

Table S4: Tukey's Honestly Significant Difference (HSD) post-hoc analysis for alanine aminotraferase (ALT) levels (U/L) in HepG2 cells.

Group 1	Group 2	Mean Difference	p-adj	Lower Bound	Upper Bound	Significant (Reject H_0)
CCl_4 and PME 10 µg/ml	CCl_4 and PME 15 µg/ml	-1.22	0.6359	-3.8454	1.4054	No
CCl_4 and PME 10 µg/ml	CCl_4 and PME 25 µg/ml	-2.08	0.1553	-4.7054	0.5454	No
CCl_4 and PME 10 µg/ml	CCl_4 and Silymarin (25 µg/ml)	0.04	1.0000	-2.5854	2.6654	No
CCl_4 and PME 10 µg/ml	CCl_4 and Vehicle	1.20	0.6506	-1.4254	3.8254	No
CCl_4 and PME 10 µg/ml	Control	-3.91	0.0032	-6.5354	-1.2846	Yes
CCl_4 and PME 15 µg/ml	CCl_4 and PME 25 µg/ml	-0.86	0.8722	-3.4854	1.7654	No
CCl_4 and PME 15 µg/ml	CCl_4 and Silymarin (25 µg/ml)	1.26	0.6065	-1.3654	3.8854	No
CCl_4 and PME 15 µg/ml	CCl_4 and Vehicle	2.42	0.0774	-0.2054	5.0454	No
CCl_4 and PME 15 µg/ml	Control	-2.69	0.0435	-5.3154	-0.0646	Yes
CCl_4 and PME 25 µg/ml	CCl_4 and Silymarin (25 µg/ml)	2.12	0.1434	-0.5054	4.7454	No
CCl_4 and PME 25 µg/ml	CCl_4 and Vehicle	3.28	0.0122	0.6546	5.9054	Yes
CCl_4 and PME 25 µg/ml	Control	-1.83	0.2503	-4.4554	0.7954	No

CCl_4 and Silymarin (25 µg/ml)	CCl_4 and Vehicle	1.16	0.6798	-1.4654	3.7854	No
CCl_4 and Silymarin (25 µg/ml)	Control	-3.95	0.0030	-6.5754	-1.3246	Yes
CCl_4 and Vehicle	Control	-5.11	0.0003	-7.7354	-2.4846	Yes

Table S5: Lactate dehydrogenase (LDH) levels (U/L) in HepG2 cells.

Group	Mean	Standard Deviation (SD)
Control	230.63	10.31
CCl_4 and Vehicle	311.03	4.82
CCl_4 and Silymarin (25 µg/ml)	246.50	5.15
CCl_4 and PME 10 µg/ml	282.54	2.60
CCl_4 and PME 15 µg/ml	261.58	3.75
CCl_4 and PME 25 µg/ml	239.26	3.71

ANOVA results for lactate dehydrogenase (LDH) levels (U/L): F-statistic = 86.20, p-value = 0.0000

Table S6: Tukey's Honestly Significant Difference (HSD) post-hoc analysis for lactate dehydrogenase (LDH) levels (U/L) in HepG2 cells.

Group 1	Group 2	Mean Difference	p-adj	Lower Bound	Upper Bound	Significant (Reject H_0)
CCl_4 and PME 10 µg/ml	CCl_4 and PME 15 µg/ml	-20.96	0.0067	-36.42	-5.50	Yes
CCl_4 and PME 10 µg/ml	CCl_4 and PME 25 µg/ml	-43.28	0.0000	-58.74	-27.82	Yes
CCl_4 and PME 10 µg/ml	CCl_4 and Silymarin (25 µg/ml)	-36.04	0.0001	-51.50	-20.58	Yes

CCl_4 and PME 10 µg/ml	CCl_4 and Vehicle	28.49	0.0005	13.03	43.95	Yes
CCl_4 and PME 10 µg/ml	Control	-51.91	0.0000	-67.37	-36.45	Yes
CCl_4 and PME 15 µg/ml	CCl_4 and PME 25 µg/ml	-22.32	0.0041	-37.78	-6.86	Yes
CCl_4 and PME 15 µg/ml	CCl_4 and Silymarin (25 µg/ml)	-15.08	0.0575	-30.54	0.38	No
CCl_4 and PME 15 µg/ml	CCl_4 and Vehicle	49.45	0.0000	33.99	64.91	Yes
CCl_4 and PME 15 µg/ml	Control	-30.95	0.0002	-46.41	-15.49	Yes
CCl_4 and PME 25 µg/ml	CCl_4 and Silymarin (25 µg/ml)	7.24	0.6292	-8.22	22.70	No
CCl_4 and PME 25 µg/ml	CCl_4 and Vehicle	71.77	0.0000	56.31	87.23	Yes
CCl_4 and PME 25 µg/ml	Control	-8.63	0.4597	-24.09	6.83	No
CCl_4 and Silymarin (25 µg/ml)	CCl_4 and Vehicle	64.53	0.0000	49.07	79.99	Yes
CCl_4 and Silymarin (25 µg/ml)	Control	-15.87	0.0431	-31.33	-0.41	Yes
CCl_4 and Vehicle	Control	-80.40	0.0000	-95.86	-64.94	Yes

Table S7: Lipid peroxidase (LPO) levels (µmoles/MDA/min/mg protein) in goat liver homogenate.

Group	Mean	Standard Deviation (SD Calculated)
Control	1.66	0.065
CCl_4	4.36	0.067
CCl_4 and PME 10 µl/ml	4.70	0.047
CCl_4 and PME 15 µl/ml	2.80	0.199
CCl_4 and PME 25 µl/ml	2.50	0.164
CCl_4 and Silymarin 10 µl/ml	2.03	0.568
PME 25 µl/ml	1.43	0.304

Table S8: Aspartate aminotransferase (AST) levels (µmoles/min/mg protein) in goat liver homogenate.

Group	Mean	Standard Deviation (SD Calculated)
Control	60.66	4.12
CCl_4	169.00	4.27
CCl_4 and PME 10 µl/ml	166.66	5.76
CCl_4 and PME 15 µl/ml	155.02	4.49
CCl_4 and PME 25 µl/ml	97.33	13.15
CCl_4 and Silymarin 10 µl/ml	65.32	6.55
PME 25 µl/ml	53.99	5.27
Silymarin 10 µl/ml	38.42	4.47

Table S9: Alanine aminotraferase (ALT) levels (µmoles/min/mg protein) in goat liver homogenate.

Group	Mean	Standard Deviation (SD Calculated)
Control	34.02	3.19
CCl_4	237.33	14.76
CCl_4 and PME 10 µl/ml	220.33	4.62
CCl_4 and PME 15 µl/ml	197.52	4.63
CCl_4 and PME 25 µl/ml	165.67	3.52
CCl_4 and Silymarin 10 µl/ml	85.67	53.35
PME 25 µl/ml	82.65	3.54
Silymarin 10 µl/ml	43.75	5.38

Table S10: Lactate dehydrogenase (LDH) levels (µmoles/min/mg protein) in goat liver homogenate.

Group	Mean	Standard Deviation (SD Calculated)
Control	12.33	2.53
CCl_4	73.65	4.31
CCl_4 and PME 10 µl/ml	67.43	2.74
CCl_4 and PME 15 µl/ml	45.41	3.13
CCl_4 and PME 25 µl/ml	23.33	1.86
CCl_4 and Silymarin 10 µl/ml	28.56	1.72
PME 25 µl/ml	9.78	0.39
Silymarin 10 µl/ml	7.75	0.40

Table S11: Analysis of variance (ANOVA) test reults for lipid peroxidase (LPO; (µmoles/MDA/min/mg protein), aspartate aminotransferase (AST; µmoles/min/mg protein), alanine aminotraferase (ALT; µmoles/min/mg protein), and lactate dehydrogenase (LDH; (µmoles/min/mg protein) levels in goat liver homogenate.

Parameter	Sum of Squares (Between Groups)	df	F-Value	p-Value
LPO	35.87	7	81.42	2.58×10^{-11}
AST	62678.20	7	189.09	3.58×10^{-14}
ALT	138834.63	7	36.47	1.14×10^{-8}
LDH	14120.11	7	327.95	4.61×10^{-16}

Table S12: Tukey's honestly significant difference (HSD) post-hoc analysis for lipid peroxidase (LPO) levels (µmoles/MDA/min/mg protein) in goat liver homogenate.

Group 1	Group 2	Mean Difference	p-Value	Lower Bound	Upper Bound	Significant ($p < 0.05$)
Control	CCl_4	-2.70	0.0001	-3.12	-2.28	Yes
Control	CCl_4 + PME 10 µl/ml	-3.04	0.0001	-3.46	-2.62	Yes
Control	CCl_4 + PME 15 µl/ml	-1.14	0.004	-1.56	-0.72	Yes
Control	CCl_4 + PME 25 µl/ml	-0.84	0.029	-1.26	-0.42	Yes
Control	CCl_4 + Silymarin 10 µl/ml	-0.37	0.478	-0.79	0.05	No
Control	PME 25 µl/ml	0.23	0.835	-0.19	0.65	No
Control	Silymarin 10 µl/ml	0.41	0.430	-0.01	0.83	No
CCl_4	CCl_4 + PME 10 µl/ml	0.34	0.502	-0.08	0.76	No
CCl_4	CCl_4 + PME 15 µl/ml	1.56	0.0001	1.14	1.98	Yes
CCl_4	CCl_4 + PME 25 µl/ml	1.86	0.0001	1.44	2.28	Yes
CCl_4	CCl_4 + Silymarin 10 µl/ml	2.33	0.0001	1.91	2.75	Yes

Table S13: Tukey's honestly significant difference (HSD) post-hoc analysis for aspartate aminotransferase (AST) levels (µmoles/min/mg protein) in goat liver homogenate.

Group 1	Group 2	Mean Difference	p-Value	Lower Bound	Upper Bound	Significant ($p < 0.05$)
Control	CCl_4	-108.34	0.0001	-117.42	-99.26	Yes
Control	CCl_4 + PME 10 µl/ml	-106.00	0.0001	-115.08	-96.92	Yes
Control	CCl_4 + PME 15 µl/ml	-94.36	0.0001	-103.44	-85.28	Yes
Control	CCl_4 + PME 25 µl/ml	-36.67	0.0001	-45.75	-27.59	Yes
Control	CCl_4 + Silymarin 10 µl/ml	-4.66	0.017	-13.74	4.42	No
Control	PME 25 µl/ml	6.67	0.061	-2.41	15.75	No
Control	Silymarin 10 µl/ml	22.24	0.0001	13.16	31.32	Yes

Table S14: Tukey's honestly significant difference (HSD) post-hoc analysis for alanine aminotraferase (ALT) levels (µmoles/min/mg protein) in goat liver homogenate.

Group 1	Group 2	Mean Difference	p-Value	Lower Bound	Upper Bound	Significant ($p < 0.05$)
Control	CCl_4	-203.31	0.0001	-217.44	-189.18	Yes
Control	CCl_4 + PME 10 µl/ml	-186.31	0.0001	-200.44	-172.18	Yes
Control	CCl_4 + PME 15 µl/ml	-163.50	0.0001	-177.63	-149.37	Yes
Control	CCl_4 + PME 25 µl/ml	-131.65	0.0001	-145.78	-117.52	Yes

Control	CCl_4 + Silymarin 10 µl/ml	-51.65	0.0001	-65.78	-37.52	Yes
Control	PME 25 µl/ml	-48.50	0.0001	-62.63	-34.37	Yes
Control	Silymarin 10 µl/ml	-9.23	0.038	-23.36	4.90	Yes

Table S15: Turkey honestly significant (LDH) post-hoc analysis for lactate dehydrogenase (LDH) levels (µmoles/min/mg protein) in goat liver homogenate.

Group 1	Group 2	Mean Difference	p-Value	Lower Bound	Upper Bound	Significant ($p < 0.05$)
Control	CCl_4	-61.32	0.0001	-66.21	-56.43	Yes
Control	CCl_4 + PME 10 µl/ml	-55.10	0.0001	-59.99	-50.21	Yes
Control	CCl_4 + PME 15 µl/ml	-33.08	0.0001	-37.97	-28.19	Yes
Control	CCl_4 + PME 25 µl/ml	-10.99	0.0001	-15.88	-6.10	Yes
Control	CCl_4 + Silymarin 10 µl/ml	-15.22	0.0001	-20.11	-10.33	Yes
Control	PME 25 µl/ml	2.55	0.645	-2.34	7.44	No
Control	Silymarin 10 µl/ml	4.58	0.308	-0.31	9.47	No
